# A study on the diagnostic value of ultrasound combined with serum MK and TSP-1 in diagnosing placental abruption

**DOI:** 10.3389/fmed.2026.1906391

**Published:** 2026-10-27

**Authors:** Yanfei Cui, Houmei Han

**Affiliations:** 1 Affiliated Hospital of Shandong Academy of Traditional Chinese Medicine, Jinan, Shandong, China; 2 Shandong Provincial Maternal and Child Health Care Hospital Affiliated to Qingdao University, Jinan, Shandong, China

**Keywords:** diagnostic accuracy, midkine, placental abruption, thrombospondin-1, ultrasound

## Abstract

**Objective:**

To explore the clinical value of combined detection of Midkine (MK) and thrombospondin-1 (TSP-1) in serum with ultrasound in the diagnosis of placental abruption.

**Methods:**

A total of 138 pregnant women with suspected placental abruption during antenatal check-ups at Shandong Provincial Maternal and Child Health Care Hospital Affiliated to Qingdao University from July 2022 to July 2025 were enrolled. They were divided into a placental abruption group (*n* = 88) and a non-placental abruption group (*n* = 50) based on whether placental abruption occurred. Both groups underwent ultrasound examination and serum MK and TSP-1 level testing. The serum MK and TSP-1 levels, ultrasound findings, and pregnancy outcomes were compared between the two groups. A combined diagnostic model was constructed using binary logistic regression in R software, and internal validation was performed using bootstrap optimism correction with 500 resamples. The combined diagnostic model performance was evaluated by ROC curve analysis, calibration curves, and decision curve analysis.

**Results:**

Serum MK and TSP-1 levels were significantly higher in the placental abruption group than in the non-abruption group (*P* < 0.05). Ultrasound findings in the placental abruption group were significantly more prevalent than in the non-abruption group (*P* < 0.05). The combined diagnostic method showed higher sensitivity, accuracy, and Youden’s index than ultrasound or serum MK/TSP-1 alone, and its AUC was significantly higher than that of each individual marker (*P* < 0.05). After bootstrap internal validation, the optimism-corrected AUC was 0.809, and the calibration error (mean absolute error) was 0.09 indicating good model performance. Pregnancy outcomes were significantly poorer in the placental abruption group.

**Conclusion:**

Ultrasound combined with serum MK and TSP-1 testing may offer potential as an adjunctive diagnostic tool to aid in the evaluation of clinically suspected placental abruption compared to single-marker approaches in this exploratory retrospective cohort.

## 1 Introduction

Placental abruption is a severe pathological process that occurs after 20 weeks of pregnancy or during the delivery period (1, 2). It is a serious complication during pregnancy (1, 2). It has been reported that the incidence of placental abruption in China ranges from 0.46% to 2.1%, while the perinatal mortality rate is as high as 10% to 20% (3). Placental abruption can lead to various adverse consequences, such as postpartum hemorrhage, coagulation dysfunction, fetal distress, and even intrauterine fetal death (3, 4). When the area of placental detachment is large and the process is rapid, the fetus will quickly lose oxygen and nutrient supply from the placenta, which may cause severe hypoxia or even death within a short period of time (5). If not diagnosed and intervened promptly, it will have a significant impact on the patients. Therefore, exploring efficient, accurate, and timely diagnostic methods is of important clinical value.

Currently, ultrasound examination is one of the commonly used methods for diagnosing placental abruption, which can directly display the separation as well as the presence of hematoma (6). However, ultrasound examination has relatively low sensitivity for placental abruption, ranging from approximately 25% to 50%, and its detection rate is limited to 25% to 60% of cases, particularly for early or concealed abruption (6). In contrast, when ultrasound findings are positive, specificity can reach 96% (7). Especially when the placenta is located on the posterior wall of the uterus, the hematoma is small or no obvious hematoma has formed, conventional gray-scale ultrasound and color Doppler ultrasound often fail to provide reliable positive signs, resulting in diagnostic delays in some patients. Therefore, relying solely on ultrasound examinations is difficult to meet the requirements for timely and accurate identification of placental abruption.

In recent years, serological markers have shown good application prospects in diagnosing pregnancy-related diseases. Midkine (MK) and Thrombospondin-1 (TSP-1) are two factors closely related to placental function and angiogenesis, and their expression levels in patients with placental abruption may be abnormal. MK plays an important role in placental development and angiogenesis. It may affect the proliferation and differentiation of placental trophoblast cells in placental abruption patients, leading to poor placental function and increasing the risk of placental abruption (8). TSP-1, as an endogenous angiogenesis inhibitor, may have changes in expression during placental abruption related to placental vascular lesions and thrombosis. Studies have shown that the expression level of TSP-1 in patients with placental abruption may be abnormal. It participates in placental abruption by regulating the function of vascular endothelial cells and promoting platelet aggregation, resulting in reduced placental blood perfusion and intravascular coagulation, and ultimately causing placental abruption (9, 10). In conclusion, the abnormal changes in serum MK and TSP-1 levels may be involved in the pathogenesis of placental abruption through different pathways. The combined detection of these two indicators is expected to serve as non-invasive biomarkers to assist in ultrasound examinations, thereby potentially improving diagnostic accuracy in clinically suspected cases, particularly when ultrasound findings are inconclusive.

Given the limitations of ultrasound examination in detecting placental abruption, especially in occult or inconclusive cases, while serum markers MK and TSP-1 play roles in placental angiogenesis, trophoblast function and coagulation pathways and show diagnostic potential, this study aims to explore the diagnostic accuracy of ultrasound combined with serum MK and TSP-1 detection for placental abruption. We intend to assess whether the combination of these two biomarkers can improve accuracy on the basis of ultrasound alone, especially for clinically challenging cases. The results may generate hypotheses for prospective evaluation of this combined strategy to potentially improve diagnostic discrimination in suspected cases, though this requires prospective validation.

## 2 Materials and methods

### 2.1 Research subjects

This retrospective study consecutively included pregnant women who received routine prenatal examinations at Shandong Provincial Maternal and Child Health Care Hospital Affiliated to Qingdao University from July 2022 to July 2025 and met the clinical criteria for suspected placental abruption. Potentially eligible patients were identified through electronic medical record review based on clinical presentation and prenatal visit documentation, rather than through prospective enrollment. Initially, 156 patients were screened. According to the pre-defined inclusion and exclusion criteria, 18 cases were subsequently excluded: 4 cases due to age ≥35 years, 5 cases due to multiple pregnancies, 3 cases due to concurrent malignant tumors, autoimmune diseases or severe infections, and 6 cases due to incomplete clinical data or interrupted follow-up. Finally, 138 patients were included, and they were divided into the placental abruption group (*n* = 88) and the non-placental abruption group (*n* = 50) based on the pathological examination results. Figure 1 shows the schematic diagram of the patient recruitment process in accordance with the STROBE guidelines. Both groups of pregnant women underwent ultrasound, serum MK, and TSP-1 level tests. The study was approved by the Ethics Committee of Shandong Provincial Maternal and Child Health Care Hospital Affiliated to Qingdao University (Ethics Number: NO. 2025-033). As this was a retrospective study, the requirement for written informed consent was waived by the Ethics Committee.

**Figure 1:**
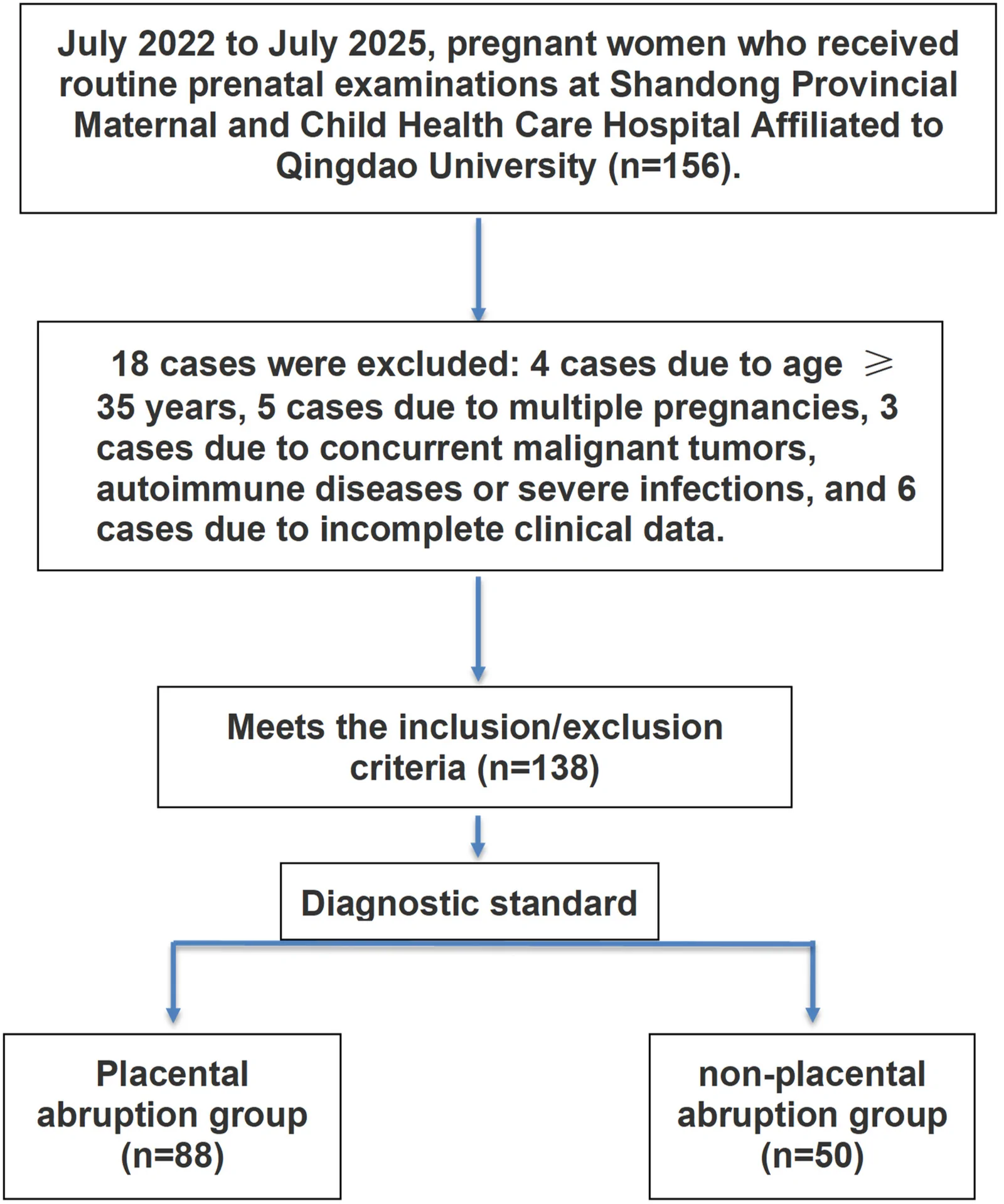
STROBE flow diagram of the study population. A total of 156 patients were screened, 18 were excluded, and 138 were included in the final analysis.

Inclusion criteria: (1) Clinically suspected placental abruption; (2) Age <35 years (According to the authoritative definitions of high-risk pregnancy by WHO and ACOG (11, 12), this group of women aged ≥35 years has clear placental vascular dysfunction and changes in expression profiles of vascular generation-related markers such as sFlt-1/PlGF and TSP-1, which may interfere with the determination of the core indicators of this study, so they were excluded). (3) Singleton pregnancy; (4) No bad living habits such as smoking, excessive drinking, or drug abuse, and no history of long-term exposure to harmful chemical substances, radiation, etc.

Exclusion criteria: (1) With hematological abnormalities, malignant tumors, autoimmune diseases, and severe infectious diseases; (2) With other reproductive system diseases; (3) With combined important organ functional disorders; (4) With contraindications for imaging examinations; (5) Taking drugs that may affect coagulation function, vascular function, etc. (6) Fetal chromosomal or genetic abnormalities identified by prenatal screening (NT ultrasound, NIPT, fetal anomaly ultrasound), invasive prenatal diagnosis when indicated, or postnatal pediatric evaluation. All cases with suspected genetic disorders were excluded.

### 2.2 Diagnostic criteria

#### 2.2.1 Clinical criteria for suspected placental abruption

According to the WHO Managing Complications in Pregnancy and Childbirth (MCPC) guidelines, a pregnant woman after 20 weeks of gestation was considered to have suspected placental abruption if she met at least two of the following criteria (13): (1) Sudden onset of persistent abdominal pain or back pain; (2) Vaginal bleeding (may be concealed or revealed); (3) Uterine tenderness or hypertonicity on palpation (in severe cases, a “woody” or “board-like” uterus); (4) Non-reassuring fetal heart rate pattern (e.g., late decelerations, prolonged deceleration, or sinusoidal pattern); (5) Unexplained preterm labor or premature rupture of membranes. In addition, the following clinical manifestations, when present, further support the suspicion: uterine hypertonicity/abnormal contractions, maternal hemodynamic instability (shock disproportionate to visible blood loss, suggesting concealed bleeding), coagulation abnormalities (including disseminated intravascular coagulation), and unexplained fetal compromise not attributable to other causes. All enrolled patients were confirmed through medical record review to have met these clinical suspicion criteria at the time of their initial presentation, prior to any imaging or serological testing being performed. These criteria were used as the basis for retrospective inclusion rather than as part of a prospective enrollment protocol.

#### 2.2.2 Diagnostic criteria for placental abruption

According to the diagnostic standards recommended (13–15), the gold standard for diagnosing placental abruption was placental pathological examination, which was performed in all 138 participants. The diagnostic criteria are as follows: Pathological diagnostic criteria (15) require the area of blood clot marks on the maternal side of the placenta to be ≥ 5 cm^2^, the abruption area to involve ≥1/4 of the total placental surface, and microscopic evidence of hemorrhage, necrosis, and fibrinoid deposition in the placental basal decidua. Patients were classified as having placental abruption if at least two of the three pathological criteria were met. We acknowledge that these pathological criteria may miss mild or early-stage abruptions, potentially leading to verification bias where true cases are misclassified as non-abruption. In addition, intraoperative findings were recorded for the 112 women who underwent cesarean delivery but were used as supplementary information rather than as a component of the reference standard.

Serum samples were collected and ultrasound examinations were performed upon admission, prior to delivery and before the pathological examination was performed. All index tests were therefore completed before the reference standard was applied, satisfying the temporal requirement for diagnostic accuracy studies.

#### 2.2.3 Ultrasound diagnostic criteria

According to the positive criteria for ultrasound diagnosis of placental abruption (7): (1) An irregular liquid-dark area (post-placental hematoma) appears between the placental base and the uterine wall, with rough edges and no or very little blood flow signal; (2) Local or diffuse thickening of the placenta, with a thickness of ≥5 cm, and an irregular contour; (3) The distance placenta-uterine wall widens, the villous plate protrudes into the amniotic cavity, and the echo of the placental substance is uneven; (4) Hidden bleeding can be observed as insufficient amniotic fluid, point-like echoes floating in the amniotic cavity, or bloody amniotic fluid. If these characteristics are met, it can be diagnosed as placental abruption (ultrasound diagnosis is positive).

#### 2.2.4 Development of the combined diagnostic model

To develop the combined diagnostic model, a binary logistic regression model was constructed with pathological diagnosis of placental abruption as the dependent variable. The independent variables were restricted to ultrasound findings (positive/negative), serum MK level, and serum TSP-1 level. The diagnosed probability derived from this model was used as the combined diagnostic index for ROC curve analysis. A positive combined diagnosis was assigned when the model-diagnosed probability exceeded the optimal cutoff value determined by maximizing the Youden index. This diagnostic model is distinct from the multivariable association model used for confounder adjustment, which is presented in Table 3.

### 2.3 Methods

(1) Comparison of general data of the two groups of pregnant women: Compare the general data of the two groups of pregnant women, including age, BMI, gestational age, number of pregnancies, number of deliveries, number of miscarriages, systolic blood pressure, diastolic blood pressure, albumin level, history of early vaginal bleeding during pregnancy, abnormal amniotic fluid, gestational diabetes, etc.
(2) Comparison of serum MK and TSP-1 levels of the two groups of pregnant women: Venous blood samples (5 mL) were collected from each participant prior to any therapeutic interventions (including antihypertensives, magnesium sulfate, corticosteroids, or anticoagulants) in the morning after an overnight fast upon admission. Blood samples were centrifuged at 1,000 × g for 15 min at room temperature, and the separated serum was aliquoted and stored at −20 °C until assay, with repeated freeze-thaw cycles strictly avoided. To ensure pre-analytical reliability of TSP-1 measurements, the following additional measures were implemented: (1) All blood samples were collected using standard serum separator tubes and processed within 2 h of collection; (2) Samples with visible hemolysis (indicated by pink or red plasma) were excluded, as inadvertent hemolysis during blood withdrawal has been shown to cause significantly elevated TSP-1 values; (3) Serum was aliquoted into multiple cryovials to avoid repeated freeze-thaw cycles—previous studies have demonstrated that human TSP-1 immunoreactivity shows no significant loss after up to three freeze-thaw cycles, and ELISA kit manufacturers recommend storage at −20 °C in small aliquots (16); (4) Samples from both groups were assayed on the same plates with inter-plate calibrators to minimize batch effects. Serum MK levels were measured using a commercial human MK enzyme-linked immunosorbent assay (ELISA) kit (Abcam, UK, catalog number: ab193761), and serum TSP-1 levels were measured using a Human Thrombospondin-1 ELISA Kit (Invitrogen, Wuhan, catalog number: BMS2100). Both assays are sandwich ELISA formats. The MK kit has a detection range of 39.1–2,500 pg/mL with a sensitivity of 14.4 pg/mL, an intra-assay coefficient of variation (CV) of 2% and an inter-assay CV of 3.9%. The TSP-1 kit (BMS2100) has a detection range of 1.17–75 ng/mL with a sensitivity of 0.044 ng/mL, an intra-assay CV of 3.4% and an inter-assay CV of 4.4%. For calibration, a four-parameter logistic (4-PL) regression model was used to fit the standard curves by plotting the mean optical density at 450 nm (OD450) against the corresponding concentrations, and sample concentrations were calculated by interpolation from the standard curve. Absorbance was read at 450 nm using a microplate reader. Serum MK concentrations, originally measured in pg/mL, were converted to ng/mL (1 ng/mL = 1,000 pg/mL) for consistent presentation with TSP-1 values.
(3) Ultrasound examination. Ultrasound examinations were performed using a GE Voluson E10 ultrasound system (GE Healthcare, USA) with a convex array probe operating at 3.5–5.0 MHz. Patients were examined in the supine position with the probe placed on the lower abdomen. A standardized multi-slice scanning protocol was performed in longitudinal, transverse, and oblique planes to comprehensively evaluate the placenta, including its location, echogenicity, retroplacental space, and the interface between the placenta and uterine wall. Placental thickness was measured at the maximal vertical dimension from the chorionic plate (fetal side) to the basal plate (maternal side) in a mid-sagittal plane; a thickness ≥5 cm was considered abnormal. The amniotic fluid volume, fetal position, and fetal biometry were also assessed. All ultrasound examinations were performed by two experienced sonographers (each with >5 years of experience in obstetric ultrasound), who independently performed the examinations and interpreted the images. In cases of disagreement, a consensus was reached through discussion. The following abnormal findings were recorded: (a) irregular anechoic or hypoechoic area between the placenta and uterine wall (suggesting retroplacental hematoma), with no or minimal blood flow signal on color Doppler; (b) local or diffuse placental thickening (≥5 cm) with irregular contour; (c) widening of the retroplacental space with protrusion of the chorionic plate into the amniotic cavity and heterogeneous placental echotexture; (d) signs of concealed bleeding, including oligohydramnios, floating echogenic particles in the amniotic cavity, or echogenic amniotic fluid. Color Doppler was used to evaluate the blood flow signals in the placental region and the umbilical artery pulsatility index. All sonographers were blinded to the serum MK and TSP-1 results, as well as to the final clinical diagnosis. Representative ultrasound images are shown in Figure 2A (placental abruption) and Figure 2B (normal placenta). See Figures 2A,B.
(4) Compare the occurrence of cesarean section, perinatal death, uteroplacental apoplexy, postpartum hemorrhage, premature birth, and disseminated intravascular coagulation in the two groups of pregnant women.

**Figure 2:**
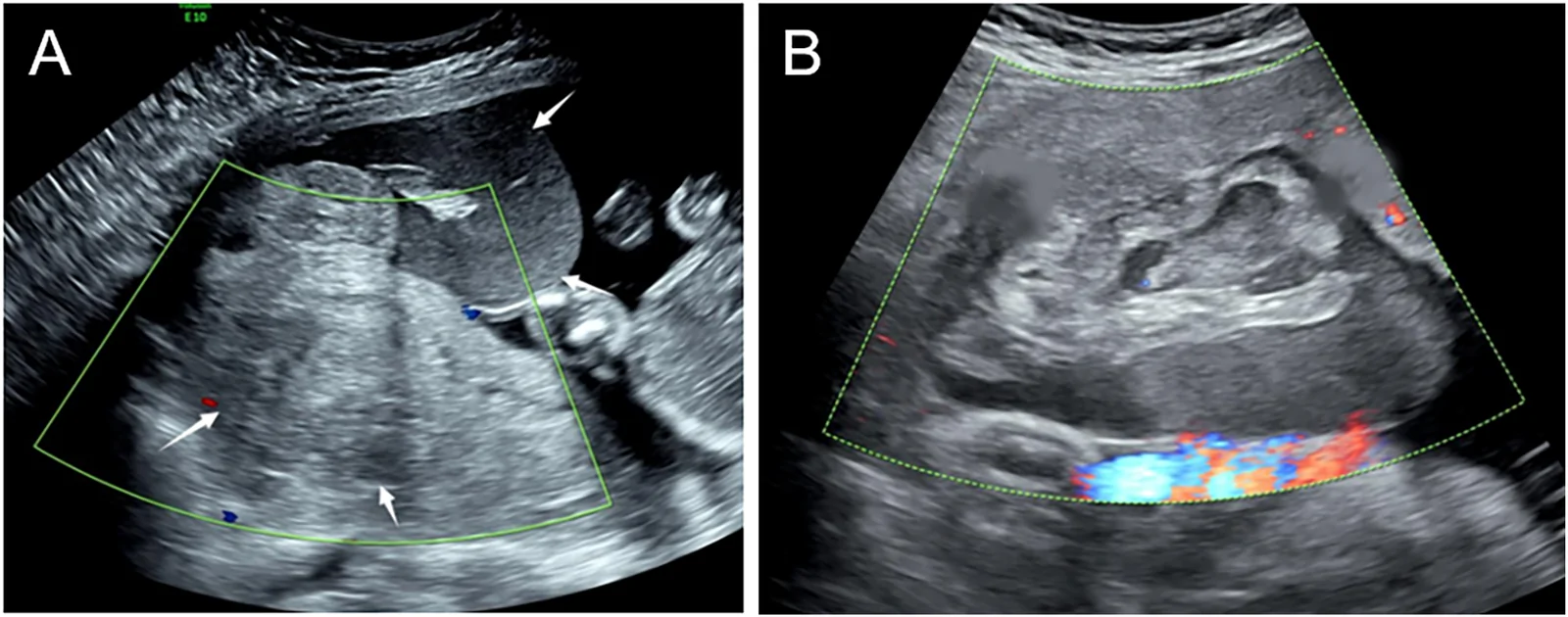
Ultrasound image comparison. **(A)** Is a patient with placental abruption; **(B)** Represents a normal patient. Panel A shows placental abruption with increased placental thickness, retroplacental hematoma, and heterogeneous echotexture (arrows). Panel B shows a normal placenta with uniform echogenicity and clear retroplacental space.

### 2.4 Statistical method

This was a retrospective study, and the sample size was determined by the available cohort rather than by a prospective power calculation. A total of 138 patients were included in the final analysis (88 in the placental abruption group and 50 in the non-abruption group). For the multivariable logistic regression model with seven predictors, the events per variable (EPV) was 88/7 ≈ 12.6, and the number of non-events also supported stable estimation of specificity. For blood pressure data, outliers were screened using the boxplot (IQR) method. Values outside Q1–1.5 ×  IQR or Q3 + 1.5 × IQR were defined as statistical outliers and marked as missing. Missing blood pressure values were imputed using multiple imputation. The data were analyzed using SPSS 23.0. Continuous variables were compared using independent-sample t-tests and expressed as mean ± SD. Normality was assessed using the Shapiro–Wilk test, and homogeneity of variances was assessed using Levene's test. If equal variances could be assumed (Levene's test *P* ≥ 0.05), the standard Student's t-test was applied; if variances were unequal (Levene's test *P* < 0.05), Welch's *t*-test with Satterthwaite approximation for degrees of freedom was used instead; Categorical variables were compared using Pearson's chi-square test when all expected cell counts were ≥5, or Fisher's exact test when any expected cell count was <5, and expressed as *n* (%). Diagnostic performance metrics were analyzed using R software (version 4.2.1). For the combined diagnostic model, a binary logistic regression model was constructed with ultrasound findings, serum MK, and serum TSP-1 as independent variables, and the diagnosed probability was used to generate the ROC curve via the pROC package, with the optimal cutoff determined by maximizing the Youden index. Internal validation was performed using optimism-corrected bootstrap resampling with 500 iterations (sampling with replacement). For each bootstrap sample, the model was refitted and evaluated on the bootstrap sample (apparent performance) and the original sample (test performance). The optimism was calculated as the difference between apparent and test performance, and subtracted from the original apparent estimate to yield the optimism-corrected estimate. Model calibration was assessed by plotting diagnosed probabilities against observed frequencies (calibration plot) and quantified using the calibration slope (ideal = 1), calibration intercept (ideal = 0), and mean absolute error (MAE). The decision curve analysis (DCA) was performed using the dcurves package in R. We compared the net benefit of the combined model against two default strategies: treat-all (assume all patients have abruption) and treat-none (assume no patients have abruption) across threshold probabilities ranging from 0% to 100%. Collinearity was evaluated using the variance inflation factor (VIF). Pairwise comparisons of ROC curves were performed using DeLong's test, with Bonferroni correction applied for multiple comparisons. A difference was considered statistically significant if *P* < 0.05.

## 3 Results

### 3.1 Comparison of general data of the two groups of pregnant women

The general data of the two groups of patients, including age, BMI, gestational age, number of pregnancies, deliveries, miscarriages, systolic blood pressure, diastolic blood pressure, albumin level, history of early vaginal bleeding during pregnancy, abnormal amniotic fluid, gestational diabetes, preeclampsia, chronic hypertension, fetal growth restriction, and placental insufficiency, showed no significant differences (*P* > 0.05). See Table 1. All patients underwent serum biomarker testing and ultrasound examination upon admission, prior to delivery and pathological confirmation.

**Table 1:** Comparison of general information of pregnant women in two groups [$\bar{x}\pm s$, *n* (%)].

Index	Placental abruption group (*n* = 88)	Non-placental abruption group (*n* = 50)	*t/χ* ^2^	*P*
Age (year)	28.51 ± 4.21	28.12 ± 4.20	0.525	0.600
BMI (kg/m^2^)	23.56 ± 3.13	23.19 ± 2.98	0.662	0.509
Gestational age (weeks)	34.23 ± 3.48	34.19 ± 2.99	0.068	0.946
Number of pregnancies (time)	2.11 ± 0.90	2.12 ± 1.11	−0.036	0.971
Number of deliveries (time)	1.67 ± 0.67	1.64 ± 0.80	0.238	0.812
Number of miscarriages (time)	0.44 ± 0.52	0.48 ± 0.54	−0.392	0.695
Systolic blood pressure (mmHg)^†^	144.96 ± 13.62	141.38 ± 12.16	1.542	0.126
Diastolic blood pressure (mmHg)^†^	90.88 ± 8.25	88.66 ± 7.89	1.543	0.121
Albumin level (g/L)	25.61 ± 4.32	25.11 ± 5.33	0.590	0.556
History of early vaginal bleeding during pregnancy	32 (36.36)	17 (34.00)	0.078	0.780
Abnormal amniotic fluid	22 (25.00)	10 (20.00)	1.518	0.218
Gestational diabetes	25 (28.41)	8 (16.00)	2.698	0.100
Preeclampsia	23 (26.14)	14 (28.00)	0.056	0.812
Chronic hypertension	26 (29.55)	15 (30.00)	0.003	0.956
Fetal growth restriction	31 (35.23)	16 (32.00)	0.145	0.703
Placental dysfunction	43 (48.86)	24 (48.00)	0.009	0.923

† Blood pressure outliers were screened using the boxplot (IQR) method.

Data are presented as mean ± SD or *n* (%).

Values outside Q1–1.5 × IQR or Q3 + 1.5 × IQR were set to missing and imputed using multiple imputation. Chronic hypertension was defined by pre-existing medical history, not by admission blood pressure alone.

### 3.2 Comparison of serum MK and TSP-1 levels between the two groups of pregnant women

The levels in the placental abruption group were significantly higher than the non-placental abruption group (*P* < 0.05). See Table 2. To assess whether MK and TSP-1 were independently associated with placental abruption after controlling for confounders, we constructed a multivariable logistic regression model including age, BMI, preeclampsia, chronic hypertension, and gestational diabetes as covariates. After adjusting for age, BMI, preeclampsia, chronic hypertension, and gestational diabetes, serum MK (aOR = 3.756, 95% CI: 1.928–7.318, *P* < 0.001) and TSP-1 (aOR = 2.489, 95% CI: 1.383–4.501, *P* = 0.003) remained independently associated with placental abruption. All VIFs were below 2, indicating no significant collinearity. See Table 3.

**Table 2:** Comparison of serum MK and TSP-1 levels in the two groups of pregnant women ($\bar{x}\pm s$, ng/mL).

Group	MK	TSP-1
Placental abruption group (*n* = 88)	1.03 ± 0.38	7.72 ± 2.24
Non-placental abruption group (*n* = 50)	0.71 ± 0.13	4.86 ± 1.19
*t*	6.920	8.373
*P*	<0.001	<0.001

Data are presented as mean ± SD.

MK: Welch's *t*-test; TSP-1: Student's t-test.

**Table 3:** Multivariable logistic regression analysis of factors independently associated with placental abruption.

Index	aOR	95% CI	*P*	VIF
Age (year)	0.903	0.785–1.021	0.132	1.118
BMI (kg/m^2^)	0.881	0.751–1.025	0.101	1.076
Gestational diabetes	1.412	0.531–3.758	0.498	1.341
Preeclampsia	1.487	0.596–3.712	0.392	1.418
Chronic hypertension	0.372	0.118–1.114	0.078	1.049
MK	3.756	1.928–7.318	<0.001	1.327
TSP-1	2.489	1.383–4.501	0.003	1.283

### 3.3 Comparison of ultrasound diagnostic results of the two groups of pregnant women

By comparing the ultrasound diagnostic results, it was found that in the group with placental abruption, the proportions of increased placental thickness (42.05%), placental edge hematoma (22.73%), irregular liquid dark area at the placental base (25.00%), and strong mixed echo (29.55%) were higher than the non-placental abruption group (4.00%, 2.00%, 0.00%, 2.00%), and the differences were statistically significant (*P* < 0.05). See Table 4.

**Table 4:** Comparison of ultrasound diagnostic results between the two groups of pregnant women [*n* (%)].

Group	Placental thickness	Placental edge hematoma	Irregular liquid dark area at the placental base	Strong mixed echo
Placental abruption group (*n* = 88)	37 (42.05)	20 (22.73)	22 (25.00)	26 (29.55)
Non-placental abruption group (*n* = 50)	2 (4.00)	1 (2.00)	0 (0.00)	1 (2.00)
*χ* ^2^	22.763	10.617	14.871	15.373
*P*	<0.001	0.001	<0.001	<0.001

### 3.4 ROC curve analysis of ultrasound and serum MK, TSP-1 alone and in combination for the diagnosis of two groups of pregnant women

The results showed that the AUC value of combined diagnosis was 0.819 (0.749–0.889), while the individual diagnoses of ultrasound and serum MK, TSP-1 were 0.671 (0.581–0.762), 0.731 (0.647–0.815), and 0.695 (0.603–0.786) respectively. The AUC value of the combined diagnosis ROC curve was greater than that of the individual diagnoses(*P* < 0.05). See Table 5 and Figure 3. Pairwise comparisons of ROC curves using DeLong's test revealed that the combined diagnosis achieved significantly higher AUC than ultrasound (*P* < 0.001), MK (*P* < 0.001), and TSP-1 (*P* < 0.001). No significant differences were observed among the individual diagnostic methods (all *P* > 0.05) (Supplementary Table S1).

**Table 5:** Diagnostic performance of the combined diagnostic model (ultrasound + MK + TSP-1) and individual markers.

Index	AUC	95%CI	Youden index	Cutoff	*P*
Ultrasound	0.671	0.581–0.762	0.343	–	<0.001
MK	0.731	0.647–0.815	0.299	0.795	<0.001
TSP-1	0.695	0.603–0.786	0.380	6.585	<0.001
Combination	0.819	0.749–0.889	0.496	0.286	<0.001

**Figure 3:**
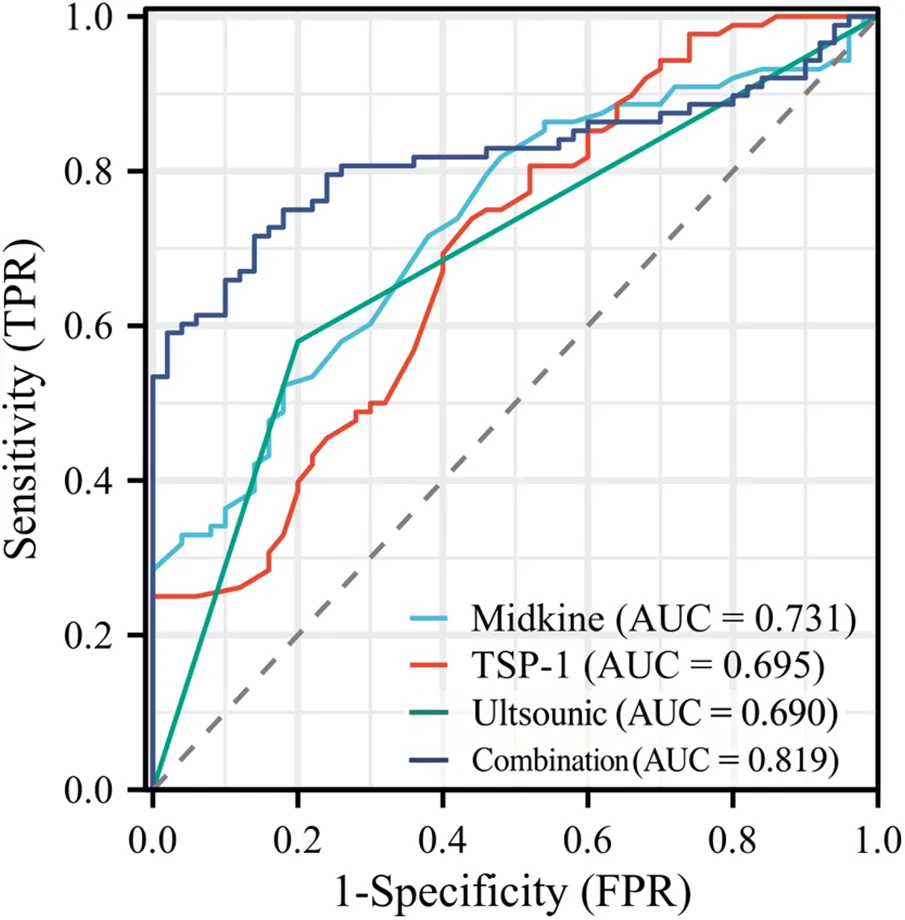
ROC curve analysis of ultrasound and serum MK, TSP-1 alone and in combination for diagnosis of two groups of pregnant women.

### 3.5 Regression equation of the combined diagnostic model

The combined diagnostic model incorporated ultrasound findings (positive = 1, negative = 0), serum MK (ng/mL), and serum TSP-1 (ng/mL) as independent variables. The logistic regression equation was: logit(P) = –5.3502 + 1.7641 × (ultrasound) + 2.2271 × (MK) + 0.8742 × (TSP-1).

The diagnosed probability of placental abruption was calculated as *P* = 1/(1 + e^(–logit(P))^). The full regression coefficients are provided in Supplementary Table S2. The model showed good discrimination with an AUC of 0.819 (Table 5).

### 3.6 Analysis of the individual and combined detection efficacy of MK and TSP-1 in ultrasound and serum

In the detection of placental abruption, the sensitivity of the combined diagnostic method, the accuracy, and the Youden index were all higher than those of the single diagnostic method. This indicates that the combined diagnosis is more effective than the single diagnosis. See Table 6 and Supplementary Table S3.

**Table 6:** Diagnostic efficacy metrics of individual markers and the combined diagnostic model.

Results	Ultrasound	MK	TSP-1	Combination
Sensitivity/%	52.27 (41.96–62.39)	73.86 (63.82–81.91)	57.95 (47.52–67.72)	79.55 (69.97–86.65)
Specificity/%	82.00 (69.20–90.23)	56.00 (42.31–68.84)	80.00 (66.96–88.75)	70.00 (56.25–80.90)
Accuracy/%	63.04 (54.74–70.64)	67.39 (59.19–74.65)	65.94 (57.70–73.32)	76.09 (68.33–82.44)
Positive predictive value	83.64 (71.74–91.15)	74.71 (64.67–82.67)	83.61 (72.39–90.85)	82.35 (72.90–89.00)
Negative predictive value	49.40 (38.91–59.94)	54.90 (41.39–67.73)	51.95 (40.97–62.75)	66.04 (52.59–77.31)

### 3.7 Internal validation of the combined diagnostic model

Internal validation was performed using optimism-corrected bootstrap resampling with 500 iterations. After bootstrap optimism correction, the optimism-corrected AUC was 0.809 (95% CI: 0.738–0.881), compared with the apparent AUC of 0.819 (optimism 0.010). At the optimal cutoff of 0.286, the optimism-corrected sensitivity and specificity were 76.69% and 67.17%, respectively (apparent sensitivity 79.55%, specificity 70.00%; optimism 2.86% and 2.83%, respectively). The calibration slope was 0.94 (95% CI: 0.71–1.14), and the calibration intercept was 0.06 (95% CI: −0.21 to 0.34), indicating good calibration with only slight overfitting and no significant systematic overestimation or underestimation of risk. The calibration curve showed good agreement between diagnosed and observed probabilities, with a mean absolute error of 0.09 after bootstrap correction. The DCA demonstrated that the combined diagnostic model provided a positive net benefit compared with treat-all and treat-none strategies across clinically relevant threshold probabilities of approximately 5% to 60%, with net benefit values ranging from 0.01 to 0.07. At a threshold probability of 20%, the net benefit was approximately 0.04. These findings support the potential clinical utility of the model for diagnostic risk stratification in suspected placental abruption, though prospective external validation is required. See Figure 4.

**Figure 4:**
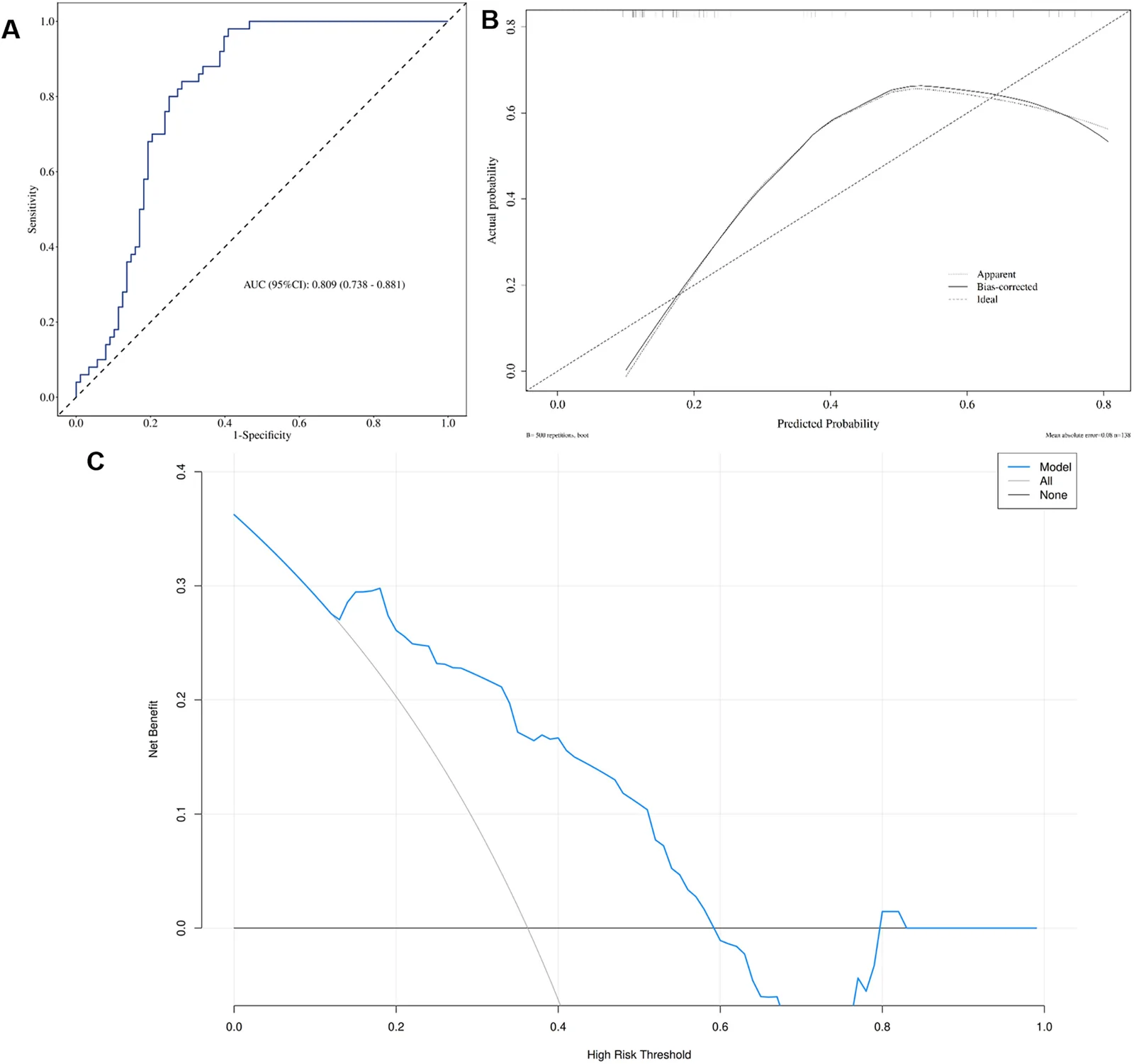
Internal validation of the diagnostic model. **(A)** ROC curve after bootstrap optimism correction (corrected AUC = 0.809, 95% CI: 0.738–0.881). **(B)** Calibration curve showing agreement between diagnosed probabilities and observed frequencies; the dashed diagonal line represents perfect calibration, and the solid line represents the fitted calibration curve (calibration slope = 0.94, intercept = 0.06, MAE = 0.09 after correction). **(C)** Decision curve analysis demonstrating net benefit across a range of threshold probabilities; the gray line represents the treat-all strategy, and the horizontal black line at y = 0 represents the treat-none strategy.

### 3.8 Comparison of pregnancy outcomes

By comparing the pregnancy outcomes, the proportions of cesarean section (*P* = 0.038), perinatal death (*P* < 0.001), uteroplacental apoplexy (*P* = 0.036), postpartum hemorrhage (*P* = 0.032), and premature birth (*P* < 0.001) in the placental abruption group were significantly higher than in the non-abruption group. The difference in disseminated intravascular coagulation (*P* = 0.055) did not reach statistical significance. See Table 7.

**Table 7:** Comparison of pregnancy outcomes between the two groups [*n* (%)].

Pregnancy outcome	Placental abruption group (*n* = 88)	Non-placental abruption group (*n* = 50)	*χ* ^2^	*P*
Cesarean section	76 (86.36)	36 (72.00)	4.302	0.038
Perinatal death	25 (28.41)	1 (2.00)	14.543	<0.001
Uteroplacental apoplexy	14 (15.91)	2 (4.00)	4.412	0.036
Postpartum hemorrhage	12 (13.64)	1 (2.00)	–	0.032
Premature birth	63 (71.59)	19 (38.00)	14.920	<0.001
Disseminated Intravascular Coagulation	11 (12.50)	1 (2.00)	–	0.055

*P* values were calculated using Pearson's chi-square test for outcomes with expected cell counts ≥5 (cesarean section, perinatal death, uteroplacental apoplexy and premature birth); Fisher's exact test was used for outcomes with expected cell counts <5 (postpartum hemorrhage and DIC).

## 4 Discussion

Placental abruption is a severe and critical emergency in the middle and late stages of pregnancy, characterized by sudden onset and rapid progression. Identification is often challenging, and misdiagnosis or delayed diagnosis frequently leads to adverse outcomes (1, 2). The current clinical gold standard is pathological examination, which has a lag (17). In addition, before the operation, the main method of diagnosis relied on ultrasound examination, but its sensitivity for early, mild cases and placental posterior wall detachment was limited, making it difficult to meet the requirements for early and precise diagnosis (17, 18). This study, through a retrospective analysis, found that the combination of ultrasound examination and serum MK and TSP-1 detection showed improved diagnostic performance, with an AUC of 0.819, compared to any single detection method in this exploratory study. The results may generate hypotheses for prospective evaluation of this combined strategy to potentially improve diagnostic discrimination in suspected cases, though this requires prospective validation.

After IQR-based screening of blood pressure outliers and multiple imputation, the mean systolic blood pressure was 144.96 ± 13.62 mmHg in the placental abruption group and 141.38 ± 12.16 mmHg in the non-abruption group, and the mean diastolic blood pressure was 90.88 ± 8.25 mmHg and 88.66 ± 7.89 mmHg, respectively. These values remained elevated in both groups, consistent with the high-risk nature of this cohort, which consisted entirely of patients with clinically suspected placental abruption. Blood pressure did not differ significantly between groups (SBP, *P* = 0.126; DBP, *P* = 0.121). Importantly, hypertension-related variables, including preeclampsia and chronic hypertension, were adjusted for in multivariable analysis, and MK and TSP-1 remained independently associated with placental abruption. The results of this study showed that the levels of MK and TSP-1 in the serum of pregnant women in the placental abruption group were significantly higher than those in the non-placental abruption group (*P* < 0.05). MK is a heparin-binding growth factor that is synthesized by trophoblast cells in the placenta and participates in regulating placental angiogenesis and trophoblast invasion (19). When placental abruption occurs, hypoxia and ischemic reperfusion injury may stimulate the trophoblast cells to over-secrete MK, thereby promoting inflammatory responses and changes in vascular permeability, and exacerbating the damage to the placental tissue. This research result is consistent with the previous reports on the increased expression of MK in pregnancy-related complications (8). It was reported that the serum MK level of patients with preeclampsia (0.54 ± 0.23 ng/mL) was significantly higher than that of the normal pregnancy control group (0.31 ± 0.19 ng/mL), and the difference was statistically significant (*P* < 0.05). TSP-1 is an important extracellular matrix protein that is mainly secreted by platelets, endothelial cells, and macrophages, and plays a key role in regulating cell adhesion, proliferation, and angiogenesis (20). In the pathological environment of placental abruption, tissue damage and platelet activation may lead to the release of TSP-1 into the blood, resulting in an increase in its serum level. Relevant studies have reported that the median serum TSP-1 level in normal pregnant mothers is 5.1 ng/mL (IQR: 2.6–7.4 ng/mL), while the TSP-1 level in the placental abruption group in this study was significantly higher than the upper limit of this reference range, consistent with the trend of increased TSP-1 in patients with pregnancy complications in the previous studies (3). The previous studies of these indicators are consistent with the changing trend of this study, enhancing the credibility of this research.

In the field of ultrasound imaging, this study identified characteristic manifestations such as thickening of the placenta, hematoma at the placental margin, irregular liquid-dark areas at the placental base, and strong mixed echoes. These findings are consistent with the ultrasound signs of placental abruption described in previous studies (21). It is worth noting that although ultrasound examination can directly display abnormal shapes and positions of the placenta, its sensitivity for early or localized placental detachment is limited (7). In this study, the sensitivity of simple ultrasound diagnosis was 52.27%, which was basically consistent with the range reported in the literature (25%–50%). This limitation arises not only due to the changing echo characteristics of the hematoma over time, but also due to factors such as the position of the placenta and the mother's body type (22).

The important finding of this study is the superiority of the combined diagnostic model strategy, which has a solid basis in pathophysiology. The essence of placental abruption is an acute injury event of the vascular network at the placental-uterine interface, and its occurrence and development follow a dynamic pathological process (23, 24). In the early stage of the disease, the rupture of blood vessels in the basal decidua of the placenta triggers localized bleeding, at this time, the changes in tissue structure are not yet captured by ultrasound imaging, but molecular-level changes have already occurred (20). The increase in serum MK levels reflects the early stress response of placental tissue to ischemia and hypoxia, while at the same time, the injury of vascular endothelium immediately activates platelets, causing them to release a large amounts of TSP-1 contents. These two biomarkers reveal the early pathological changes of placental abruption from different perspectives (8): MK represents the dysfunction of trophoblast cells and inflammatory activation, while TSP-1 reflects vascular endothelial injury and thrombosis tendency (3, 25). Therefore, the changes in serum MK and TSP-1 levels, which occur earlier in time than the detectable morphological changes by ultrasound, provide additional diagnostic information in the workup of clinically suspected cases. As the pathological process progresses, a hematoma gradually forms and expands, at this time, changes can be captured by ultrasound (26–28). The single diagnostic method has obvious limitations: although serum markers can sensitively reflect early molecular events, they cannot provide intuitive information on anatomical localization and degree of injury; ultrasound imaging can accurately display structural abnormalities, but is insufficient in sensitivity for initial microvascular injury and early biochemical changes (6). The advantage of the combined diagnostic model strategy lies in integrating information from different dimensions of function and structure, micro and macro, and constructing a multi-level, mutually verifying diagnostic system. This strategy may conceptually simulate a diagnostic process in which serum markers could raise suspicion, followed by ultrasound for localization and severity assessment; however, this framework is preliminary and requires prospective evaluation. When ultrasound, serum MK, and TSP-1 are combined, the diagnostic sensitivity increases to 79.55%, the accuracy is 76.09%, and the Youden index is 0.496, its overall performance showed improvement compared to any single detection method. We acknowledge that the diagnostic value demonstrated in this study applies to patients already under clinical suspicion for placental abruption. The biomarkers were measured at the time of admission after clinical suspicion had been raised; therefore, our findings support the role of MK and TSP-1 as confirmatory or adjunctive tools when ultrasound is inconclusive, rather than as pre-symptomatic screening tests. Whether these biomarkers can detect abruption before clinical presentation requires longitudinal studies in asymptomatic at-risk populations. This result suggests a more comprehensive reflection of the pathophysiological process compared with single markers, though this interpretation is exploratory and warrants further mechanistic studies.

This study also found that the incidence of adverse pregnancy outcomes in the placental abruption group was generally higher than that in the non-placental abruption group. Specifically, the cesarean section rate in the placental abruption group was 86.36% (76/88), which was higher than 72.00% (36/50) in the control group (*P* = 0.038); the perinatal mortality rate was 28.41% (25/88), which was much higher than 2.00% (1/50) in the control group (*P* < 0.001); the incidence of postpartum hemorrhage was 13.64% (12/88), which was higher than 2.00% (1/50) in the control group (*P* = 0.032); the preterm birth rate was 71.59% (63/88), which was significantly higher than 38.00% (19/50) in the control group (*P* < 0.001). Although the incidences of disseminated intravascular coagulation (12.50% vs. 2.00%, *P* = 0.055) were numerically elevated in the abruption group, the differences did not reach statistical significance. These results indicate that the risk of severe adverse outcomes for both the mother and the fetus in cases of placental abruption is substantially increased. Consistent with previous literature reports, the perinatal mortality rate of placental abruption is 20% to 35%, approximately 15 times that of those without abruption (29); in patients with placental abruption accompanied by pathological changes in placental tissue, the incidence of combined severe neonatal outcomes is also significantly higher than that of those without pathological changes (*P* = 0.005) (30). This evidence indicates that the severity of placental abruption is positively correlated with adverse maternal and infant outcomes.

Literature reports indicate that through fetal heart monitoring combined with ultrasound for early diagnosis of placental abruption and timely termination of pregnancy, the incidence of uteroplacental apoplexy, the incidence of postpartum complications, and the mortality rate of perinatal infants have all significantly decreased (29). Among them, the mortality rate of perinatal infants can be reduced to 8.30%, significantly lower than 35.0% in the group that did not receive timely diagnosis (1). In this study, the AUC, sensitivity and Youden index of the multimodal combined diagnostic model strategy of ultrasound combined with serum MK and TSP-1 showed better apparent performance than each individual detection method in this study. Bootstrap internal validation further demonstrated a well-calibrated model with an optimism-corrected AUC of 0.809 (95% CI: 0.738–0.881), corrected sensitivity of 76.69% and specificity of 67.17% at the optimal cutoff, a calibration slope of 0.94, calibration intercept of 0.06, and a mean absolute error of 0.09, indicating good calibration with only slight overfitting and no significant systematic bias. The decision curve analysis showed a positive net benefit across threshold probabilities of approximately 5% to 60%, supporting the potential clinical utility of the combined approach for diagnostic risk stratification in suspected cases. The combined diagnostic model strategy may provide useful information for diagnostic risk stratification in suspected cases—for pregnant women with atypical ultrasound manifestations but abnormal serological indicators, increased clinical vigilance and appropriate management should be considered to avoid adverse pregnancy outcomes. The integration of multi-dimensional information may potentially allow for enhanced diagnostic workup in suspected cases and more refined risk assessment, which might contribute to improved outcomes, but this remains to be confirmed in prospective investigations. This study also found that the incidence of adverse pregnancy outcomes in the placental abruption group significantly increased, including cesarean section, perinatal death, uteroplacental apoplexy, postpartum hemorrhage, and premature birth. These results are consistent with the trends of multiple reports on complications of placental abruption (31), further highlighting the importance of timely diagnosis and intervention.

Based on the results of this study, we suggest that a strategy combining ultrasound with serum MK and TSP-1 warrants further prospective investigation as a potential adjunctive tool for pregnant women with high-risk factors for placental abruption. For high-risk pregnant women, abnormal combined detection indicators might warrant increased clinical awareness even if the ultrasound changes are atypical; while for cases where ultrasound suggests suspicion but serum markers are normal, unnecessary excessive treatment might be avoided. However, given the exploratory nature of this single-center retrospective study and the fact that all performance estimates were derived from the development dataset without external validation, these findings should be interpreted with caution and should not be construed as evidence sufficient for clinical implementation. Prospective external validation in independent cohorts is mandatory before any clinical application.

Several limitations of this study should be acknowledged. First, as a single-center retrospective study with a relatively small and imbalanced sample (88 vs. 50), selection bias and limited generalizability cannot be excluded, and the potential for residual confounding remains despite multivariable adjustments for major clinical covariates. Second, serum MK and TSP-1 were measured only once upon admission, and their specificity requires further evaluation; these biomarkers reflect disease status at presentation rather than dynamic changes throughout pregnancy, so their true early diagnostic accuracy remains unclear. Third, the exclusion of women aged ≥35 years and those with fetal chromosomal abnormalities may limit generalizability to these subgroups. Fourth, the reference standard itself may have introduced bias. The pathological criteria for placental abruption (requiring at least two of three features: blood clot ≥5 cm^2^, abruption area ≥1/4 of placental surface, and microscopic hemorrhage/necrosis/fibrinoid deposition) are inherently strict, which may lead to verification bias by misclassifying mild or early-stage abruptions into the non-abruption group. Such misclassification could potentially inflate the apparent diagnostic performance by excluding ambiguous cases from the case group. Additionally, since all enrolled patients were clinically suspected of placental abruption at presentation, the results may not be generalizable to asymptomatic or low-risk populations (spectrum bias). Fifth, the optimal cutoff values were derived in the same dataset without external validation, which may lead to overfitting and overestimation of diagnostic performance; external validation in independent cohorts is essential before clinical application. Although we used a parsimonious model with seven predictors (EPV ≈ 12.6), the limited sample size affects the precision of estimates, and larger prospective studies are needed to validate our findings and explore the longitudinal dynamics of MK and TSP-1 throughout pregnancy. Additionally, while we excluded visibly hemolyzed samples and processed all specimens within 2 h of collection to minimize pre-analytical variability, subclinical hemolysis, which has been reported to elevate TSP-1 values, cannot be entirely excluded, and the long-term stability of TSP-1 at −20 °C over three years warrants further evaluation.

## 5 Conclusion

In this exploratory retrospective study, ultrasound examination combined with serum MK and TSP-1 detection demonstrated promising apparent diagnostic performance for placental abruption in patients with clinically suspected disease, with an AUC of 0.819. This multimodal strategy may have potential as an adjunctive diagnostic tool to aid in the evaluation of clinically suspected placental abruption, but all findings are preliminary and require external validation. Serum MK and TSP-1, as potential biomarkers, warrant further investigation in prospective studies to confirm their clinical utility and to explore their pathophysiological roles in placental abruption.

## Data Availability

The raw data supporting the conclusions of this article will be made available by the authors, without undue reservation.
