# Supplementary material for: A study on the diagnostic value of ultrasound combined with serum MK and TSP-1 in diagnosing placental abruption
Source: Front Med (Lausanne). 2026 Oct 27;13:1906391. doi: 10.3389/fmed.2026.1906391 (PMC13748395; doi:10.3389/fmed.2026.1906391)
Supplement: Supplementary file 1 [file Supplementaryfile1.docx]

Table S1. Pairwise comparisons of ROC curves using DeLong’s test

| Comparison | AUC Difference | 95% CI | *P* |
| --- | --- | --- | --- |
| Combination vs. Ultrasound | 0.148 | 0.100–0.196 | <0.001 |
| Combination vs. MK | 0.088 | 0.044–0.132 | <0.001 |
| Combination vs. TSP-1 | 0.124 | 0.078–0.170 | <0.001 |
| MK vs. Ultrasound | 0.060 | 0.000–0.120 | 0.051 |
| TSP-1 vs. Ultrasound | 0.024 | –0.027–0.075 | 0.362 |
| MK vs. TSP-1 | 0.036 | –0.021–0.093 | 0.215 |

Note: *p* values in Table S1 are two-sided unadjusted P values from DeLong’s test. Bonferroni correction was applied for six pairwise comparisons, and the Bonferroni-adjusted significance threshold was *p*<0.0083. After Bonferroni correction, the combined model remained significantly superior to ultrasound, MK, and TSP-1 (all adjusted *p*<0.006); no significant differences were observed among the three single markers (all adjusted *p*>0.05).

Table S2. Regression coefficients of the combined diagnostic model

| Comparison | β Coefficient | SE | Wald χ² | *P* | OR | 95% CI |
| --- | --- | --- | --- | --- | --- | --- |
| Intercept | –5.3502 | 1.0972 | 23.78 | <0.001 | — | — |
| Ultrasound (positive vs. negative) | 1.7641 | 0.5402 | 10.66 | 0.0011 | 5.84 | 2.05–17.32 |
| MK (per ng/mL) | 2.2271 | 1.0196 | 4.77 | 0.0289 | 9.28 | 1.31–75.49 |
| TSP-1 (per ng/mL) | 0.8742 | 0.4517 | 3.74 | 0.0531 | 2.4 | 0.99–5.83 |

Table S3. Contingency tables for each diagnostic method

| Diagnostic method | Cutoff | TP (n=88) | FP (n=50) | FN | TN |
| --- | --- | --- | --- | --- | --- |
| Ultrasound | Positive findings (qualitative) | 46 | 9 | 42 | 41 |
| MK (ng/mL) | >0.795 | 65 | 22 | 23 | 28 |
| TSP-1 (ng/mL) | >6.585 | 51 | 10 | 37 | 40 |
| Combined model | >0.286 (probability) | 70 | 15 | 18 | 35 |

Note: Cutoff values were derived from Table 5 by maximizing the Youden index. All performance metrics in Table 6 were calculated from these counts;
